# Optical detection of radon decay in air

**DOI:** 10.1038/srep21532

**Published:** 2016-02-12

**Authors:** Johan Sand, Sakari Ihantola, Kari Peräjärvi, Harri Toivonen, Juha Toivonen

**Affiliations:** 1 STUK - Radiation and Nuclear Safety Authority, P.O. Box 14, FI-00881 Helsinki, Finland; 2 Tampere University of Technology, Department of Physics, P.O. Box 692, FI-33101 Tampere, Finland

## Abstract

An optical radon detection method is presented. Radon decay is directly measured by observing the secondary radiolumines cence light that alpha particles excite in air, and the selectivity of coincident photon detection is further enhanced with online pulse-shape analysis. The sensitivity of a demonstration device was 6.5 cps/Bq/l and the minimum detectable concentration was 12 Bq/m^3^ with a 1 h integration time. The presented technique paves the way for optical approaches in rapid radon detec tion, and it can be applied beyond radon to the analysis of any alpha-active sample which can be placed in the measurement chamber.

Radon gas is released in soil as a result of radioactive decay of uranium and thoron series. As a radioactive noble gas, radon emanates easily through porous ground to housings and is responsible for 42% of the annual radiation dose of population in the world^1^. It is widely observed that exposure to radon leads to increased risk of lung cancer^2^,^3^. Radon and some of the daughter atoms decay by emitting alpha particles which have short range in air but high damage potential if absorbed in living cells. Radon progenies are easily adhered to surfaces and therefore, the upper respiratory tract is exposed to the highest radiation dose. Due to its carcinogenic nature, radon monitoring is required in risk areas.

Radon levels are typically measured by leaving a piece of special film in a room for a fixed period of time, and the number of alpha particles incident on the film is later counted in a laboratory analysis. This approach provides a reliable and low-cost estimate of the average radon level in the premises but it is not suited to online monitoring applications. In contrast, a fast response is required in the fields of mining industry, uranium exploration, and in verification of radon repairs. Continuous radon monitoring can also be used as a warning system for earthquakes which are known to increase radon levels shortly prior to the event^4^,^5^. Currently, detectors employing ionisation chambers, semiconductor sensors, or zinc-sulphide scintillation (Lucas) cells are often used for these applications^3^.

The absorption of alpha particles in air induces secondary radioluminescence light which can be utilized for remote detection of alpha decay^6^. The light is generated by radiative relaxation of nitrogen molecules, excited by secondary electrons. The conversion efficiency from kinetic energy into optical radiation is 19 photons per each MeV of energy released in air^7^. This corresponds to approximately 100 photons when a single ^222^Rn nucleus releases all of the 5.6 MeV decay energy into air. Most of the photons are observed in the near UV region between 300 nm and 400 nm^8^. The increased range and multiplication of signal carriers are the key benefits of an optical alpha particle detection method.

This work presents the principle and first results of an optical radon measurement. The feasibility of the technique is proven using a demonstration device which is applied to a step-response test and to a longer field test to observe daily variation of radon concentration at an office property. Furthermore, the optical detector is calibrated against an established commercial detector. The technique enables direct radon detection with exceptionally large active volume and high efficiency.

## Method

The optical radon measurement is based on simultaneous detection of multiple secondary photons from the same decay event. Since the photons are generated along the alpha particle track and emitted isotropically, it is beneficial to a have a measurement volume with highly reflective walls. This enhances detection probability of a single photon by allowing multiple reflections before absorption. The ideal shape of the volume is a sphere since it has the greatest volume-to-surface-area ratio, which minimizes absorption of alpha particles into walls. For these reasons, an integrating sphere was used in the measurement setup. The sphere selected for this work (SPH-8-3 AdaptaSphere, Labsphere) has a diameter of 20 cm and it is coated with Spectraflect, which is a BaSO_4_ -based diffusive reflector. It has an estimated reflectance of 97% for the nitrogen emission.

A pump was used to continuously circulate air through the sphere with a measured flow rate of 1.4 l/min. A HEPA (high efficiency particulate) filter was placed between the pump and the sphere to remove radon progenies and dust from the inlet air. This ensured that the ambiguous radon-daughter equilibrium could not interfere with signal level. Since only radon could enter the detector, it is assumed that all ^222^Rn decays in the sphere are followed by two additional alpha decays of the two short-lived daughter atoms, namely ^218^Po and ^214^Po. The detection efficiency for daughter decay is limited to 50%, provided that they are adhered to the sphere surface and scintillation properties of BaSO_4_ are negligible.

**Figure 1:**
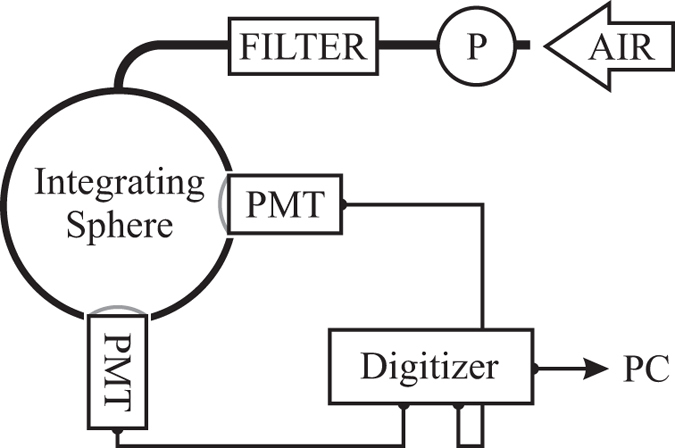
Optical radon detection setup. A pump (P) forces air into the detection volume through a filter which removes radon progenies from the incoming air. The sample leaves the detector through the PMT ports.

The coincident detection of the secondary photons was conducted with two photomultiplier tubes (PMTs) (9829QB, ET Enterprises). They were selected to provide a high detection efficiency with the large domed windows (46 mm diam. active area), which were sandblasted to further enhance sensitivity. The PMTs were operated in the photon counting regime with a gain of 10^7^, at voltages specified by the manufacturer. A schematic representation of the experimental setup is depicted in Fig. 1.

A fast digitizer (DT5751, Caen) was used to record the events in list mode with 1 ns time-stamp resolution. The device features an online coincidence detection system and a software package for pulse shape discrimination (PSD), which is typically used for neutron-gamma discrimination^9^. This is based on charge integration in two time windows of different lengths. The PSD figure is calculated from the integrated charges with equation


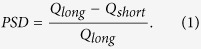


Both of the integration windows start 1 ns after the trigger signal (threshold 19.5 mV, coincidence window 32 ns) which is the minimum allowed gate offset of the digitizer. The length of the short integration window was set to 7 ns, while the long integration window was 50 ns throughout this work. The values were selected to give clear separation of alpha particles from background events.

The key concept of pulse shape discrimination is that one PMT can detect several secondary photons from the same alpha decay event, when an integrating sphere is used as detection volume. Importantly, the photons will arrive at different times since they are emitted at different times and they can travel long distances in the sphere before detection. The flight time of an alpha particle is approximately 5 ns^10^ and most of the photons are emitted within few ns after excitation due to short lifetime of the excited states^8^. However, the most significant pulse stretching arises from the random path length that photons travel in an integrating sphere. The flight time of a photon can be estimated from the sphere multiplier^11^ (M) and average path length^12^ (L) in the sphere


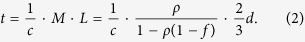


Here, *c* is the speed of light, *ρ* is the average sphere reflectance for nitrogen emission, *f* is the fraction of port area to sphere surface area (0.031), and *d* is the diameter of the sphere. By substituting the relevant values, and assuming that the absorption of near UV light is negligible in air, the equation shows that the average lifetime of a photon in the sphere is 7 ns.

## Results

The optical radon detector was applied to several tests in the ground floor of a university building in Tampere, Finland. The property is ventilated during extended office hours only, which leads to a significant rise in radon levels during nights and weekends. This natural radon occurrence and its daily variation was utilized in this work.

**Figure 2:**
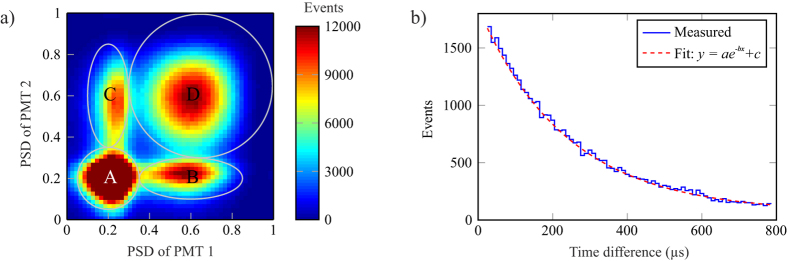
(**a**) Distribution of coincident pulse shapes over two week measurement period. Radon events accumulate to regions BCD while type A signals are mostly single photoelectron pulses. The peak value in region A is 45 000. (**b**) Time difference of subsequent coincidence events. The fitting verifies that the half-life is 164.3 μs, as expected for ^214^Po.

The UV signal is categorized on the basis of pulse-shape. The PSD values of coincident PMT signals are presented in a two-dimensional histogram in Fig. 2a, which contains all events that were recorded during a two week measurement period. Notably, the histogram reveals that pulses accumulate in four main regions, which are named ABCD. Each of these regions responds to changes in radon concentration but the signal-to-background ratio is the best for type D signal. In this region, both PMTs detect several photons so that 40% of total charge is in the short integration window. These pulses can reduce to regions B and C if one detector captures only one photoelectron or if multiple photons arrive simultaneously. Type A signals consist of single photoelectron events related to random coincidences, beta particles and gamma rays, which have a lower light yield than alpha particles. Here, BCD signals are selected to represent radon events.

The transient response of a radon detector can be limited by the two alpha-emitting daughters (^218^Po and ^214^Po) following radon in the uranium series. The first daughter atom after radon decay is ^218^Po and it reaches secular equilibrium with ^222^Rn within minutes (T_1/2_ of ^218^Po is 3.1 min^13^). However, ^214^Po requires several hours before equilibrium is reached and therefore, it should be separated from the previous two to achieve the best possible time response for the detection.

The current design enables the measurement of ^214^Po contribution by observing the successive beta and alpha emission of ^214^Bi and ^214^Po. In this decay chain, a beta particle of ^214^Bi is followed by an alpha particle of ^214^Po, with a half-life of 164 μ*s*^14^,^15^. Using temporal and pulse energy discrimination, the decay of ^214^Po can be reliably identified with an efficiency of 2.5%. This is verified with a histogram representing the distribution of time differences of consecutive coincidence events in Fig. 2b. The data set allows the determination of ^214^Po half-life with a high precision and the obtained value of 164.3 μ*s* is in agreement with a recent result of 164.2 (6) μ*s*, also measured using the latest digital electronics^15^.

**Figure 3:**
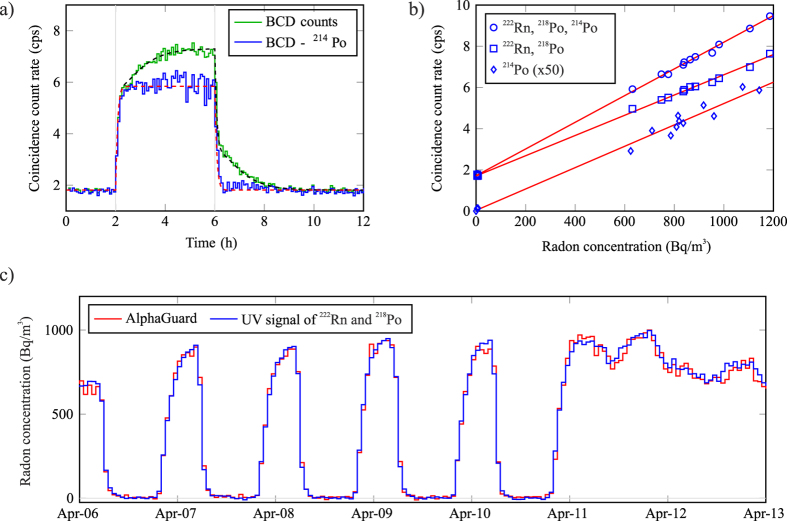
(**a**) Step response test with artificial air and ambient radon concentration of 870 Bq/m^3^. The start and end of the radon air feed are indicated with vertical lines. The modelled response is shown with a dashed line for both cases, and the experimental data points are averaged for 5 minutes. (**b**) Calibration of the UV detector with an AlphaGuard radon monitor in steady radon concentrations. The signal of ^214^Po is multiplied by a factor of 50 to enhance readability. (**c**) The evolution of UV signals during the in-field experiment. The observed radon signal follows the schedule of the ventilation system, which is active only during extended working hours. The radon concentration is logged with the AlphaGuard and the UV signal of ^222^Rn and ^218^Po is calibrated using the data of AlphaGuard. The results are presented with a 1 h integration time.

The step response of the detector was investigated during a high radon level (weekend) in the laboratory, using radon-less artificial air as a zero reference. The experiment was started with a two-hour baseline measurement with artificial air, as shown in Fig. 3a. Then, the detector was supplied with an ambient radon sample (870 Bq/m^3^ on average) for four hours to allow equilibrium formation. Lastly, the flow of artificial air was restored for six hours. The acquired step response is shown with and without the contribution of ^214^Po to highlight the necessity of ^214^Po subtraction in rapid measurements.

The experimental data is accompanied by modelled response curves in Fig. 3a. The model is based on the half-lives of radon progenies and it is fitted to the total BCD count data using the detection efficiencies for radon and daughter atoms as free parameters. The calculation shows that a good match is achieved when the detection efficiency of radon decay is 0.7 while both alpha emitting daughter atoms are detected with a reduced efficiency of 0.4 due to absorption into the sphere walls. The results were verified with a Monte Carlo model which showed that exactly the same fractions of radon and daughter alpha particles are fully absorbed into air in a detector of this size and geometry.

The performance of the optical detector was evaluated with a side-by-side test with an AlphaGuard radon monitoring system (Saphymo GmbH), which is based on detection with an ionisation chamber^16^. The AlphaGuard was set to record the radon concentration in the laboratory with 1 h integration cycle and the data were used for the calibration of the optical detector. It also logged temperature, humidity and pressure data which were close to 22 °C, 20% and 1000 mbar during the time of measurements. The calibration points were selected to be during late afternoon and early morning hours, when the radon concentration is at its most stable. The observed radon activities varied from below 20 Bq/m^3^ up to peak levels exceeding 1000 Bq/m^3^, which were reached during the nights. Using the obtained reference points, a calibration curve for the gross coincidence counts of BCD type signals was acquired, and one additional calibration was performed for the same data where the contribution of ^214^Po was subtracted. The gross sensitivity of the device was 6.5 cps/Bq/l, while ^222^Rn and ^218^Po alone yielded 4.9 cps/Bq/l, as shown in Fig. 3b. The respective detection limits were calculated using Currie’s method with a confidence level of 95%^17^. The minimum detectable concentration (MDC) under stable background conditions can be expressed as


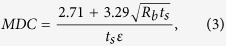


where *R*_*b*_ is the background count rate, *t*_*s*_ is the sample counting time, and *ε* is the detection efficiency of decay events per becquerel of radon in one cubic meter (i.e. the slope of linear regression in Fig. 3b). The determined MDC value for one hour measurement was 12 Bq/m^3^ for the gross signal and 15 Bq/m^3^ for ^222^Rn and ^218^Po alone. The reliability of the radon detection is further verified in Fig. 3c where the data of the AlphaGuard and the calibrated UV signal of ^222^Rn and ^218^Po are in excellent agreement. It can be also noted that the hour-to-hour fluctuation of the UV signal is minimal.

## Discussion

The main difference between the presented and established techniques is that a very high signal count rate can be achieved. Therefore, the method is not limited by statistical uncertainty of signal, which is the case with many radon detection techniques. However, the background signal is higher than in carefully designed Lucas cells and ionisation chambers and therefore, the full potential of the large active volume is not unveiled, when very low radon concentrations (below 12 Bq/m^3^) are of interest. Although the detection limits of leading commercial techniques (1–2 Bq/m^3^)^16^,^18^ were not currently reached, the performance is already more than adequate for online monitoring of radon. Secondly, the slowly changing signal of ^214^Po can be omitted to achieve readings without waiting for equilibrium formation within the detector, which is the prevalent approach with Lucas cell designs. For these reasons, the technique shows promise for applications where radon needs to be measured rapidly with small relative uncertainty. It should be also noted that the maximal measurable radon concentration can be at least several MBq/m^3^ since air is a very fast scintillator and the secondary photon burst of an alpha particle is shorter than 100 ns even in a large integrating sphere.

The presented device can also be applied beyond radon to surface activity determination of alpha- or beta-emitting samples by placing them into the measurement chamber. The obtained 1 h detection limit of 12 Bq/m^3^ for radon equals to an alpha activity of 0.05 Bq in the detection volume, which suggests that the typical definitions of alpha contamination (0.4 Bq/cm^2^ for low toxicity emitters and 0.04 Bq/cm^2^ for all others^19^) are within reach of the presented detection technique. As a practical example, clinical swab samples could be screened down to 0.4 Bq level within a minute with the current device, and by scaling the design, even hand-held tools could be rapidly checked for surface contamination. This could extend the capabilities of current small item monitors^20^ to include alpha detection, which would be of interest to the nuclear industry. The benefits of the optical approach with respect to conventional techniques are that an integral measurement is performed within the detection volume while no direct interaction with an alpha particle is required for the detection. These factors enable alpha contamination screening of objects with complex geometries in specifically designed chambers, as long as sufficient photon detection efficiency is ensured with large area photon counters.

## Conclusions

A method to directly detect radon decay via radioluminescence photons of air is reported. A demonstration device was developed and its performance was bench-marked with an established commercial detector. The obtained results show that the characteristic optical pulse shape of a radon decay in a highly reflective enclosure can be used to achieve a reliable radon detection in indoor conditions. Additionally, the detection of beta particles was verified and utilized in the identification of ^214^Bi - ^214^Po decay to enhance the response for rapid changes in radon concentration. In conclusion, the optical approach is attractive for real-time monitoring, since a large active volume can be used with high efficiency for direct detection of radon decay in air.

## Additional Information

**How to cite this article**: Sand, J. *et al*. Optical detection of radon decay in air. *Sci. Rep.* **6**, 21532; doi: 10.1038/srep21532 (2016).
