# Erratum: Optical detection of radon decay in air

**DOI:** 10.1038/srep24468

**Published:** 2016-04-22

**Authors:** Johan Sand, Sakari Ihantola, Kari Peräjärvi, Harri Toivonen, Juha Toivonen

Scientific Reports 6: Article number: 2153210.1038/srep21532; published online: 02122016; updated: 04222016

The HTML version of this Article contained a typographical error in the volume number ‘6’, which was incorrectly given as ‘5’. This has now been corrected.

In addition, there were typographical errors in the Abstract.

“Radon decay is directly measured by observing the secondary radiolumines cence light that alpha particles excite in air,”

now reads:

“Radon decay is directly measured by observing the secondary radioluminescence light that alpha particles excite in air,”

“The presented technique paves the way for optical approaches in rapid radon detec tion,”

now reads:

“The presented technique paves the way for optical approaches in rapid radon detection,”

These errors have now been corrected in the PDF and HTML versions of the Article.

